# Transport of pseudothermal photons through an anharmonic cavity

**DOI:** 10.1038/s41598-021-87536-w

**Published:** 2021-04-15

**Authors:** Dmitriy S. Shapiro

**Affiliations:** 1 Dukhov Research Institute of Automatics (VNIIA), Moscow, Russia 127055; 2 Department of Physics, National Research University Higher School of Economics, Moscow, Russia 101000; 3 Laboratory of Superconducting Metamaterials, National University of Science and Technology MISiS, Moscow, Russia 119049; 4 V. A. Kotel’nikov Institute of Radio Engineering and Electronics, Russian Academy of Sciences, Moscow, Russia 125009

**Keywords:** Quantum optics, Single photons and quantum effects, Qubits, Single photons and quantum effects, Theoretical physics

## Abstract

Under nonequilibrium conditions, quantum optical systems reveal unusual properties that might be distinct from those in condensed matter. The fundamental reason is that photonic eigenstates can have arbitrary occupation numbers, whereas in electronic systems these are limited by the Pauli principle. Here, we address the steady-state transport of pseudothermal photons between two waveguides connected through a cavity with Bose–Hubbard interaction between photons. One of the waveguides is subjected to a broadband incoherent pumping. We predict a continuous transition between the regimes of Lorentzian and Gaussian chaotic light emitted by the cavity. The rich variety of nonequilibrium transport regimes is revealed by the zero-frequency noise. There are three limiting cases, in which the noise-current relation is characterized by a power-law, $S\propto J^\gamma$. The Lorentzian light corresponds to Breit-Wigner-like transmission and $\gamma =2$. The Gaussian regime corresponds to many-body transport with the shot noise ($\gamma =1$) at large currents; at low currents, however, we find an unconventional exponent $\gamma =3/2$ indicating a nontrivial interplay between multi-photon transitions and incoherent pumping. The nonperturbative solution for photon dephasing is obtained in the framework of the Keldysh field theory and Caldeira-Leggett effective action. These findings might be relevant for experiments on photon blockade in superconducting qubits, thermal states transfer, and photon statistics probing.

## Introduction

Experimental and theoretical studies of out of equilibrium cavity and circuit QED have shown remarkable progress during the last decade^1–5^. This research area covers a diverse class of driven-dissipative phenomena and quantum phase transitions^6–9^. There, observation of photon-photon correlations and quantum state transfer becomes possible in hybrid systems^10–14^ where transmission lines are coupled to nonlinear quantum oscillators, such as superconducting transmon qubits or anharmonic cavities. In particular, the photon-photon interaction can be probed in the photon blockade effect as suggested in Ref.^15^, an optical counterpart of the Coulomb blockade in electronic devices. This is an intriguing phenomenon where a driven quantum anharmonic oscillator emits anticorrelated photon ’trains’ indicating their sub-Poissonian statistics^16–18^.

In this work, we explore the transport of incoherent photons through the cavity with anharmonicity (Kerr energy) smaller than the excitation frequency and bandwidth of the input signal. This is a bosonic counterpart of thermal or voltage biased electronic level with Coulomb interaction^19,20^. Our findings are motivated mostly by experiments on photons statistics^21–23^ and thermal states propagation^24^ that are relevant for various applications such as a microwave non-classical light emission^25^, thermometry^26^, and quantum states transfer^27^.

The moderate anharmonicity does not lead to a well-developed photon blockade, *i.e.*, photon number in the cavity is proportional to the input drive and there is no saturation of the photon current. Nevertheless, it is known that even weak anharmonicity results in the antibunching of photons and their negative correlations^28,29^. In our studies, we find that wideband incoherent pumping induces bunching of photons. We predict a transition from Lorentzian to Gaussian pseudothermal light, as follows from second-order intensity correlator $g^{(2)}$. However, the emitted light possesses a partial coherence. This results in intriguing behavior in the integral characteristics of the emitted (transmitted) photons such as their zero-frequency noise, written *S*. Contrary to $g^{(2)}$ that resolves short timescales, *S* is provided by photon counting during long time intervals. The unusual noise-current relations derived here represent an interplay between photon-photon interaction and strong incoherent drive which brings the system far from equilibrium.

The aim of this work is twofold. First, we analyze nonequilibrium noise, Fano factor, decoherence, and transmission spectrum of the cavity photons. We consider a wide range of behaviors ranging from the single-particle transfer with Breit-Wigner transmission to a many-body regime with unconventional shot noise. The second purpose is methodological. We apply the Keldysh path integral technique^30,31^, and adopt the concept of dissipative Caldeira-Leggett action^32,33^ to a driven oscillator with Bose-Hubbard interaction. Although these methods found their application in condensed matter theory a long time ago, in the cavity and circuit QED, they have started to gain attention much later. Nowadays, applications of Keldysh formalism to quantum optics is an active area of research^4,34–37^. In our methodology, a nonperturbative solution for quasi-classical fluctuations, which takes into account many-body effects and decoherence of transmitted photons is proposed.

**Figure 1:**
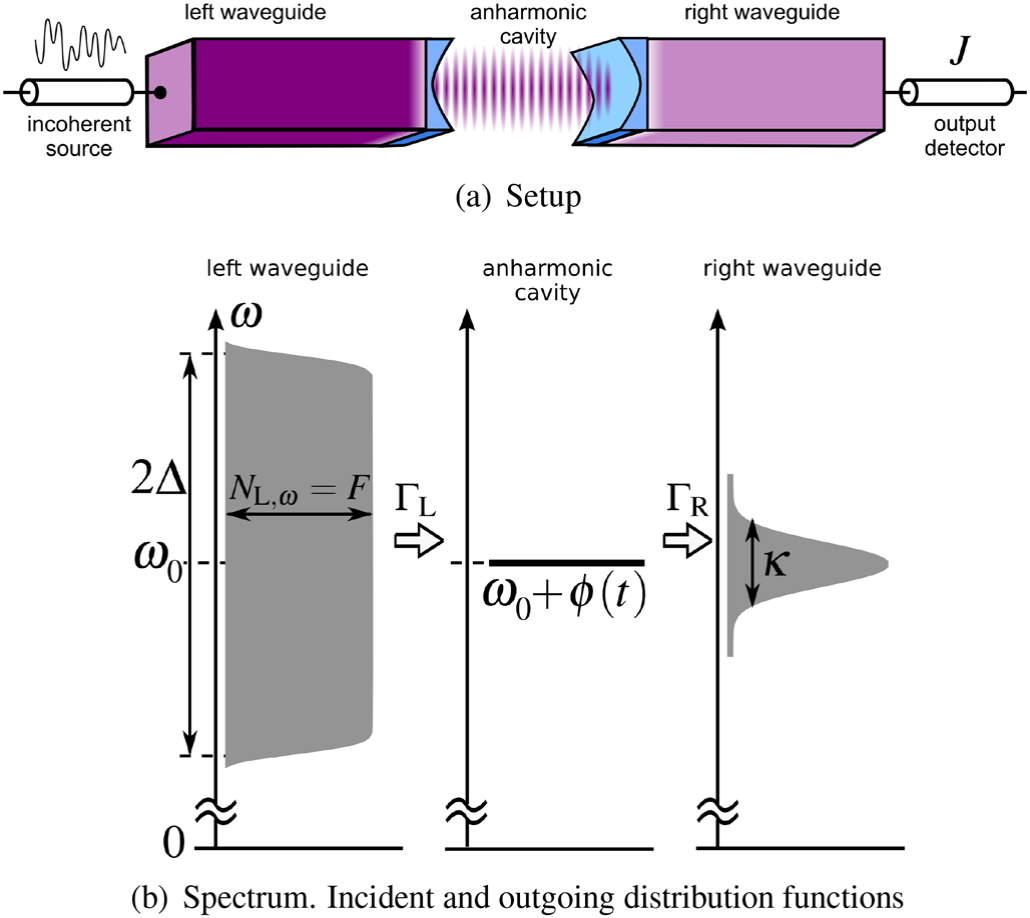
(**a**) Sketch of the setup. The incoherent drive source emits photons into the left waveguide. The difference in filling color transparencies stands for a difference in light intensities. The standing wave between blue mirrors is the cavity mode. The current *J* is measured in the output detector. (**b**) The spectrum of the incident and outgoing photon states. Incident photons have flat spectrum $N_{\mathrm{L,\omega }}=F$ of the bandwidth $\Delta$ (gray color), nonequilibrium outgoing distribution function have Gaussian shape of width $\kappa$. Fluctuating potential $\phi (t)$ is induced by the Bose-Hubbard interaction.

The sketch of the hybrid system under consideration is shown in Fig. 1(a); it is implemented as two open waveguides coupled through an anharmonic cavity^15^. Alternatively, this can be a circuit QED setup, similar to that reported in Ref.^38^, where a multilevel transmon qubit is coupled to superconducting transmission lines.

In Fig. 1(a), the left waveguide is connected to a source of thermal photons, while outgoing light is measured by a photon detector in the right waveguide. The distribution function of incident states in the left waveguide is assumed flat $N_{\mathrm{L},\omega }= F$ in the frequency range $\omega \in [\omega _0-\Delta ; \ \omega _0+\Delta ]$ and zero otherwise, as shown in Fig. 1; $\omega _0$ is the cavity mode frequency and $\Delta$ is the source linewidth. The outgoing states in the right waveguide can have Lorentzian or Gaussian nonequilibrium distribution functions. The Gaussian distribution is determined by a width $\kappa$ depending nontrivially on *F*.

The number of photons *F* in a particular incident mode can be less or greater than unity. We assume the following ordering of the relevant frequencies

1 $$\begin{aligned} \omega _0\gg \Delta \gg \varepsilon , \Gamma _{\mathrm{L,R}} , \kappa \ , \end{aligned}$$

where $\varepsilon$ is the anharmonicity, $\Gamma _{\mathrm{L}}$ and $\Gamma _{\mathrm{R}}$ are relaxation rates due to the coupling with the left and the right waveguides, and their sum $\Gamma =\Gamma _{\mathrm{L}}+\Gamma _{\mathrm{R}}$ determines the bare relaxation rate. (We set Planck constant $\hbar =1$ hereafter.) The condition (1) is motivated by typical parameters of nonlinear optical cavities^16^ where the anharmonicity is induced by a coupling of the cavity mode with a trapped atom. Also, this condition is accessible in superconducting systems based on transmon qubits coupled to transmission lines^14,17,18^. According to (1), antiresonant processes (counter-rotating wave) are neglected and the quantum dynamics is determined by the following *U*(1) Hamiltonian with continuous symmetry,

2 $$\begin{aligned} {{\hat{H}}}= {{\hat{H}}}_{\mathrm{ac}}+{{\hat{H}}}_{\mathrm{L}}+{{\hat{H}}}_{\mathrm{R}}+{{\hat{H}}}_{\mathrm{tL}}+{{\hat{H}}}_{{\mathrm{tR}}} \ . \end{aligned}$$

The first term in (2) is ${{\hat{H}}}_{\mathrm{ac}} = \omega _0{{\hat{a}}}^\dagger {{\hat{a}}} +\varepsilon {{\hat{a}}}^\dagger {{\hat{a}}}({{\hat{a}}}^\dagger {{\hat{a}}}-1)$; it governs cavity field dynamics. ${{\hat{a}}}^\dagger$ and ${{\hat{a}}}$ are, respectively, boson creation and annihilation operators acting in a basis of Fock states $|n\rangle$ with different photon numbers, and the nonlinearity $\varepsilon$ defines Bose-Hubbard interaction strength. ${{\hat{H}}}_{\mathrm{L}}=\sum _k E_{\mathrm{L},k} {{\hat{b}}}^\dagger _k {{\hat{b}}}_k$ and ${{\hat{H}}}_{\mathrm{R}}=\sum _ p E_{\mathrm{R},p} {{\hat{c}}}^\dagger _p {{\hat{c}}}_p$ describe photon dynamics in the left and right waveguides, respectively; ${{\hat{b}}}^\dagger _k$, ${{\hat{b}}}_k$ and ${{\hat{c}}}^\dagger _p$, ${{\hat{c}}}_p$ are creation and annihilation operators acting in spaces of modes labeled by a quasimomentum *k* and *p* in a respective waveguide. Couplings between the cavity and waveguide modes are assumed weak, hence, the respective Hamiltonians are postulated in the form corresponding to the rotating wave approximation, ${{\hat{H}}}_{\mathrm{tL}}=t_{\mathrm{L}} \sum _k {{\hat{b}}}_k^ \dagger {{\hat{a}}} + ({\mathrm{H.c.}})$ and ${{\hat{H}}}_{{\mathrm{tR}}}=t_{\mathrm{R}} \sum _p {{\hat{c}}}_p ^ \dagger {{\hat{a}}}+ (\mathrm{H.c.})$, where $t_{\mathrm{L}}$ and $t_{\mathrm{R}}$ are coupling amplitudes. Broad spectra of $E_{\mathrm{L},k}$ and $E_{\mathrm{R},p}$ determine densities of states, $\nu _{\mathrm{L}}$ and $\nu _{\mathrm{R}}$, at the cavity mode $\omega _0$ and relaxation rates, $\Gamma _{\mathrm{L}}=\pi \nu _{\mathrm{L}} |t_{\mathrm{L}}|^2$ and $\Gamma _{\mathrm{R}}=\pi \nu _{\mathrm{R}} |t_{\mathrm{R}}|^2$.

The measured photon current is defined as the average number of photons $n_{t_0}$ that have passed during the measurement time $t_0$, $J=\frac{1}{t_0}\langle {{\hat{n}}}_{t_0}\rangle$. Brackets denote an average for a quantum mechanical operator $\hat{{\mathcal{O}}}$ with a nonequilibrium density matrix ${{\hat{\rho }}}$, $\langle {\hat{{\mathcal{O}}}}\rangle ={{\mathrm{Tr}}}[{\hat{{\mathcal{O}}}}{\hat{\rho}}]$. The trace is calculated with the help of the nonequilibrium Keldysh technique and path integrals over complex boson fields. For the limit (1), the wideband drive induces the current $J=2F\frac{\Gamma _{\mathrm{L}}\Gamma _{\mathrm{R}}}{\Gamma }$ that does not depend on the interaction $\varepsilon$. The average photon number in the cavity, $\langle {{\hat{a}}}^\dagger {{\hat{a}}}\rangle =F \frac{ \Gamma _{\mathrm{L}} }{ \Gamma }$, does not saturate as *F* increases. This value of $\langle {{\hat{a}}}^\dagger {{\hat{a}}}\rangle$ might be associated with effective temperature $T_{\mathrm{eff}}\sim F \frac{ \Gamma _{\mathrm{L}} }{ \Gamma }\omega _0$, however, the incident photons are not distributed by the Bose-Einstein law and the cavity has non-Gibbs density matrix.

Zero frequency noise of the photon current is defined as the second cumulant of counted photons during $t_0$ interval, $S=\frac{1}{t_0}\left( \langle n_{t_0}^2\rangle -\langle n_{t_0}\rangle ^2\right)$. Below we show that the noise-current relation, *S*(*J*), and transmission probability function $T_\omega$, which appears in a photonic counterpart of the Landauer formula, depend nontrivially on $\varepsilon$. These quantities, as well as $g^{(2)}$-correlator and Fano factor, demonstrate the richness of Lorentzian-to-Gaussian crossover in a pseudothermal light.

## Results

### Non-equilibrium effective action

**Figure 2:**
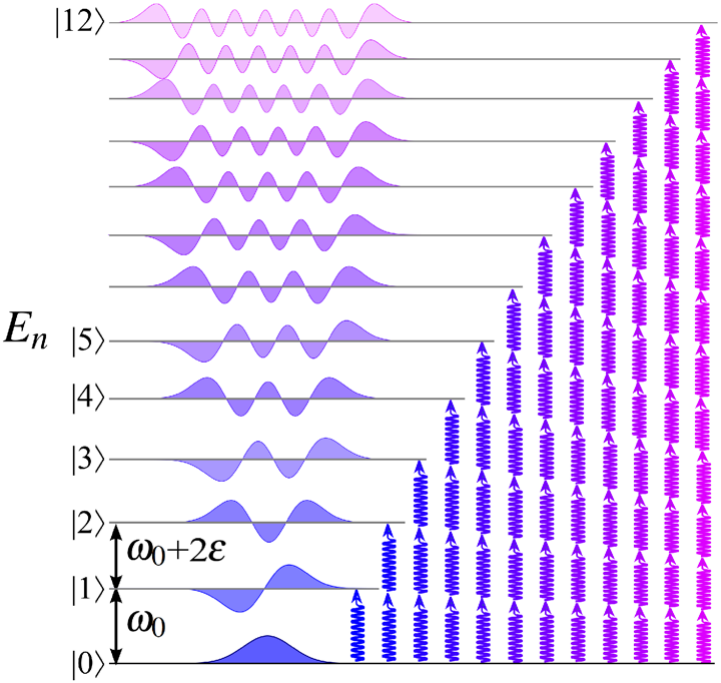
Sketch of multi-photon transitions in the cavity with a negative anharmonicity $\varepsilon <0$. Eigenstates $| n\rangle$ have non-equidistant spectrum $E_n=\omega _0 n +\varepsilon n(n-1)$ where *n* is the respective photon number. A multi-photon transition from $|0\rangle$ to $|n\rangle$ is accompanied by the absorption of *n* photons of the frequency $\omega _n=\omega _0+\varepsilon (n-1)$. The decrease of $\omega _n$ with *n* for $\varepsilon <0$ is illustrated by a change of color from blue to magenta.

We reduce the description to the Keldysh effective action ${\mathbf {S}}_{\mathrm{eff}}[\phi ]$ for the Hubbard-Stratonovich real field $\phi (t)$. It decouples the Bose-Hubbard interaction term. The dynamics of $\phi (t)$ determines the decoherence of transmitted photons. A particular configuration of $\phi (t)$ has a contribution $\sim e^{i{\mathbf {S}}_{\mathrm{eff}}[\phi ]}$ in the partition function. The dynamics of $\phi (t)$ is influenced by different multi-photon transitions in the cavity shown in Fig. 2. The particle number operator ${{\hat{n}}}={{\hat{a}}}^\dagger {{\hat{a}}}$ does commute with the cavity Hamiltonian, $[{{\hat{H}}}_{ac},{{\hat{n}}} ]=0$. Hence, its Fock states $|n\rangle$ are the same as those of the harmonic oscillator, *i.e.*, they can be represented through the canonical coordinate and Hermite polynomials. The wavefunctions in canonical coordinates should not be confused with multi-mode field configurations in the real space. Contrary to harmonic oscillator, the eigenvalues spectrum of ${{\hat{H}}}_{\mathrm{ac}}$ is a non-equidistant, $E_n=\omega _0 n+\varepsilon n(n-1)$. It is shown that a description of multi-photon transitions in the nonlinear single-mode cavity is reduced to an effective theory for $\phi (t)$. It has a stochastic behavior, which resembles Kubo model^39^ of an oscillator with random modulated frequency. For the sake of compactness, the results of this Section are presented for the symmetric setup with $\Gamma _{\mathrm{L}}=\Gamma _{\mathrm{R}}=\Gamma /2$. We find that the stationary part of $\phi (t)$ is a nonequilibrium saddle point of the action. Its value defines a shift of the cavity mode frequency by the occupation number *F* and the anharmonicity, $\omega _{\mathrm{ac}}= \omega _0+\frac{1}{2}(F-2)\varepsilon$. A nonperturbative solution for the decoherence follows from Gaussian theory for fluctuations near the saddle point. In our approach, we distinguish the fields $\phi _+(t )$ and $\phi _-(t)$ residing on the upward and backward branches of the Keldysh contour ${\mathcal{K}}$. Hence, the stochastic and quantum phases are introduced as $\Phi (t) =\frac{1}{2}\int ^t ( \phi _+ (t') + \phi _-(t') )dt'$ and $\varphi (t) =\frac{1}{2}\int ^t ( \phi _+ (t') - \phi _-(t') )dt'$, respectively. Their dynamics is governed by the action of Caldeira-Leggett type^32,33^,

3 $$\begin{aligned} i{\mathbf {S}}_{\mathrm{CL}}[ \Phi ,\varphi ] = \int \frac{i\omega ^2}{\varepsilon } \Phi _{-\omega } \varphi _{\omega }\frac{d\omega }{2\pi } \ + \ \frac{i}{2}\int \frac{\omega ^2d\omega }{2\pi } \begin{bmatrix} \Phi _{-\omega }&\varphi _{-\omega } \end{bmatrix} \begin{bmatrix} 0&& \alpha ^A_\omega \\ \\ \alpha ^R_\omega && i\alpha ^K_\omega \end{bmatrix} \begin{bmatrix} \Phi _{\omega } \\ \\ \varphi _{\omega } \end{bmatrix} \ , \end{aligned}$$

where Fourier transformed phases read as $\Phi _{\omega }=\int \Phi (t)e^{i\omega t} dt$ and $\varphi _{\omega }=\int \varphi (t)e^{i\omega t} dt$. The first term is the ’kinetic’ part, whereas the second term is Caldeira-Leggett dissipative part. It is written as a matrix with Keldysh causality structure.

In the equilibrium situation, the many-body density matrix is $\hat{\rho } = \frac{1}{{{\text{Tr}[e^{{ - i\beta \hat{H}}} ]}}}e^{{ - i\beta \hat{H}}}$, where $\beta$ is the inverted temperature. The fluctuation-dissipation theorem holds in this case. It states that the Keldysh component $\alpha ^K_\omega$ and retarded (advanced) components $\alpha ^{R(A)}_\omega$ in the effective action (3) are related as $\alpha ^K_\omega =(\alpha ^R_\omega -\alpha ^A_\omega )(1+2N_{\mathrm{B}},\omega )$, where $N_{\mathrm{B,\omega }}=\frac{1}{2}\coth \frac{\beta \omega }{2}-\frac{1}{2}$ is bosonic occupation number. In our situation with flat distribution functions, we find that dissipative terms vanish in the limit of large $\Delta$, $\alpha ^R_\omega =(\alpha ^A_\omega )^*=\frac{ 4i}{\pi \Delta ^3} \varepsilon \Gamma F^2$. Oppositely, the fluctuational Keldysh term does not vanish and depends non-linearly on the occupation number *F*, $\alpha ^K_\omega = 4 \frac{ \Gamma F (F+2)}{ 4 \Gamma ^2+\omega ^2 }$. The distribution function in the anharmonic cavity is found, $N_{\mathrm{ac,\omega }}=\frac{1}{\Gamma }(\Gamma _{\mathrm{L}}N_{\mathrm{L}} ,\omega +\Gamma _{\mathrm{R}}N_{\mathrm{R,\omega }})$. The inequality $\alpha ^K_\omega \ne (\alpha ^R_\omega - \alpha ^{A}_\omega )(1+2N_{\mathrm{ac, }}\omega)$ demonstrates a break of the fluctuation-dissipation theorem in our nonequilibrium regime.

### Decoherence

Caldeira-Leggett action is reduced to a Langevin equation^40^ $\frac{d}{dt} \Phi (t)= \varepsilon \xi (t)$ for the stochastic phase of transmitted photons. The random force in the r.h.s. has the following correlator, $\langle \xi (t)\xi (0)\rangle =\frac{1}{4}\alpha ^K(t)$, where $\alpha ^K (t)= F(F+2) e^{-2 \Gamma |t|}$ is the time-resolved Keldysh part from (3). The non-Gaussian effects of the noise can be sensitive to the dynamics of $\varphi$; this is beyond the scope of our consideration. The non-Gaussian effects might be important in the limit of large $\varepsilon >\omega _0$ when the anharmonic cavity becomes a two-level system.

The decoherence of photons is determined by the envelope $z(t)=\langle e^{i\Phi (0)-i\Phi (t)}\rangle =e^{-D(t)}$ where $D(t)=\frac{1}{4}\int ^t_0\int ^{t'}_0\alpha ^K(t'')dt''dt'$ is the phase autocorrelation function. It reads

4 $$\begin{aligned} z(t) = \mathrm{exp}\Big [- \frac{\kappa ^2}{2\Gamma ^2}(2\Gamma t + e^{-2 \Gamma |t|}-1 )\Big ]. \end{aligned}$$

The same structure of a correlator appears in the Kubo model. There is Gaussian decay at short timescale with the rate $\kappa = \frac{1}{2}\varepsilon \sqrt{F(F+2)}$, which increases with the anharmonicity and is nonanalytic by *F* due to the square root.

The behavior of *z*(*t*) plays a central role in our findings because it determines all characteristics of the outgoing light. We note that the Gaussian decay of the envelope appears in the spin-boson model with the sub-Ohmic dissipative environment with $1/\omega$ spectrum^41^. The effective sub-Ohmic spectrum of phase fluctuations in our problem is due to multi-photon transitions.

### Intensity correlator

The second-order correlator of outgoing photons, which can be probed in Hanbury-Brown and Twiss interferometry^42^, is

5 $$\begin{aligned} g^{(2)}(t)=1+z^2(t)e^{-2\Gamma t}. \end{aligned}$$

Thus, the emitted signal is chaotic pseudothermal light that can be Lorentzian or Gaussian depending on the anharmonicity $\varepsilon$ and mean photon current. Namely, the Lorentzian light with $g^{(2)}(t)\approx 1+e^{-2 \Gamma t}$ occurs at $J\ll J^*_\varepsilon$, whereas the Gaussian, $g^{(2)}(t)=1+e^{-2 \kappa ^2 t^2}$, at $J\gg J^*_\varepsilon$ is found. The value of $J^*_\varepsilon$ is related to the ratio between the anharmonicity and relaxation as $J^*_\varepsilon =\frac{\Gamma }{2}\Big (\sqrt{1+4\frac{\Gamma ^2}{\varepsilon ^2}}-1 \Big )$.

The correlator at zero time $g^{(2)}(0)>1$ is related to the bunching and $g^{(2)}(0)<1$ to the antibunching of emitted photons^43^. In our case, $g^{(2)}=2$ that is consistent with that pseudothermal photons are bunched. This occurs because the incoherent drive with a flat spectrum resembles the high-temperature limit of Bose-Einstein distribution where photon number fluctuations in a single mode are proportional to mean photon number squared.

However, the presence of coherence in the emitted light suppresses positive correlations. As shown below this is reflected in the emergence of the shot noise and in the suppression of the Fano factor. This is also indicated by the fast decay of $g^{(2)}(t)$ to the unity, which is associated with a coherent light. The decay occurs at the timescale $t\sim 1/\kappa$, which becomes very short at large *F* and $\varepsilon$.

### Transmission spectrum

It is found that the single-photon Breit-Wigner transmission function $\tau _\omega = \frac{ \Gamma ^2}{(\omega - \omega _{\mathrm{ac}})^2+\Gamma ^2 }$ (exactly-solvable $\varepsilon =0$ limit) is modified by the stochastic phase as $T_{\omega }=\int \limits ^{\infty }_{-\infty } z(t) \tau (t) e^{i \omega t } dt$. Here, $\tau (t)$ is time-resolved $\tau _\omega$. Similarly to $g^{(2)}$, the Lorentzian transmission spectrum $\tau _\omega$ is changed to Gaussian at $J\gg J^*_\varepsilon$ which reads

6 $$\begin{aligned} T_\omega =\frac{\sqrt{\pi }\Gamma }{2 \kappa } \exp \left( \!-\frac{(\omega -\omega _{\mathrm{ac}})^2}{4\kappa ^2 } \right) \ . \end{aligned}$$

This is a nonperturbative result with respect to the interaction and incoherent drive. This transmission describes nonequilibrium and many-body photon transfer.

**Figure 3:**
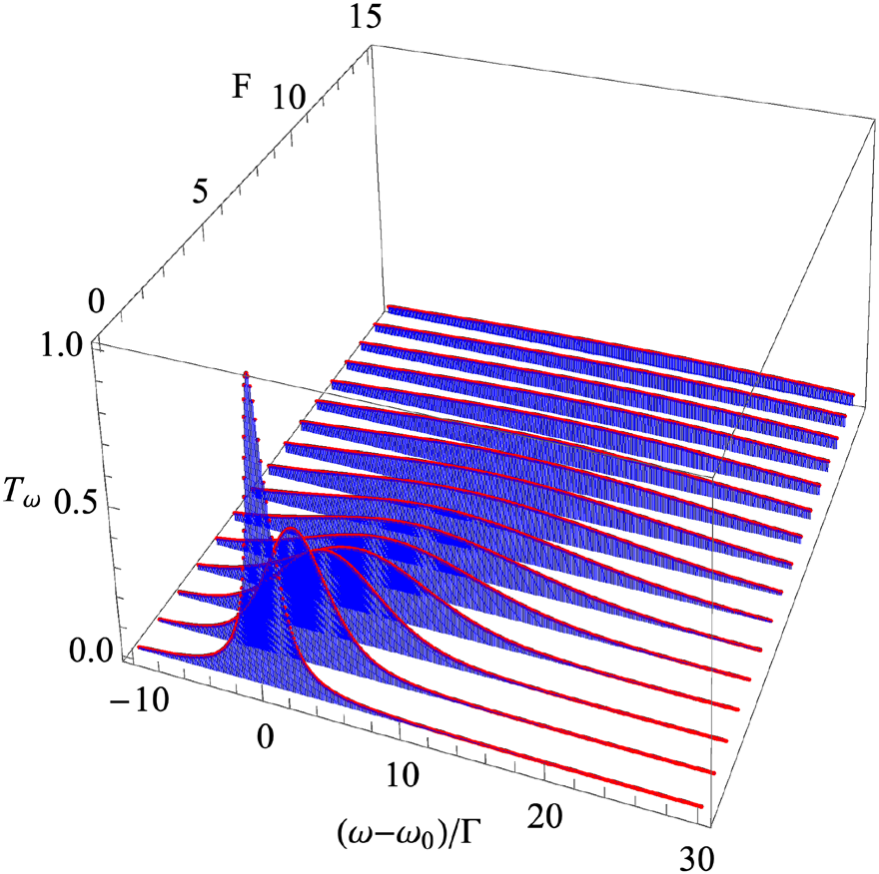
Transmission spectrum $T_\omega$ as a function of the frequency and *F*. The curve at equilibrium regime $F=0$ represents Lorentzian $\tau _\omega$ of the width $\Gamma$. The increase of *F* turns the nonequilibrium regime on and $T_\omega$ become Gaussian with $\kappa > \Gamma$. Maxima of $T_\omega$ at each *F* are located at shifted frequency $F\varepsilon /2$; here, $\Gamma _{\mathrm{L}}=\Gamma _{\mathrm{R}} =\Gamma /2$ and $\varepsilon /\Gamma =2$.

The crossover between Lorentzian and Gaussian spectra in $T_\omega$, when *F* increases, is shown in Fig. 3; results are obtained after numerical integration. It should be noted that the photon-photon interaction redistributes the spectral weight of $T_\omega$ around $\omega _{\mathrm{ac}}$, keeping its integral value constant. The latter explains that fact that the anharmonicity does not change the current in the wideband input drive regime.

### Noise

Correlations in the output field are determined by $g^{(2)}(t)$. A relevant information is given by a short timescale of ${t_0}\ll 1/\Gamma$. Distinctly, the zero-frequency noise^44^ contains information about the long timescale, $t_0\gg 1/\Gamma$. The indicated change of the asymptotic behaviors in *z*(*t*) or $T_\omega$ is reflected in the richness of a low-frequency noise-current relation *S*(*J*).

The Lorentzian emitted light shows the quadratic noise-current relation,

7 $$\begin{aligned} S_{\mathrm{therm}} =\frac{J^2}{2\Gamma } \ , \quad J\ll J^*_\varepsilon . \end{aligned}$$

**Figure 4:**
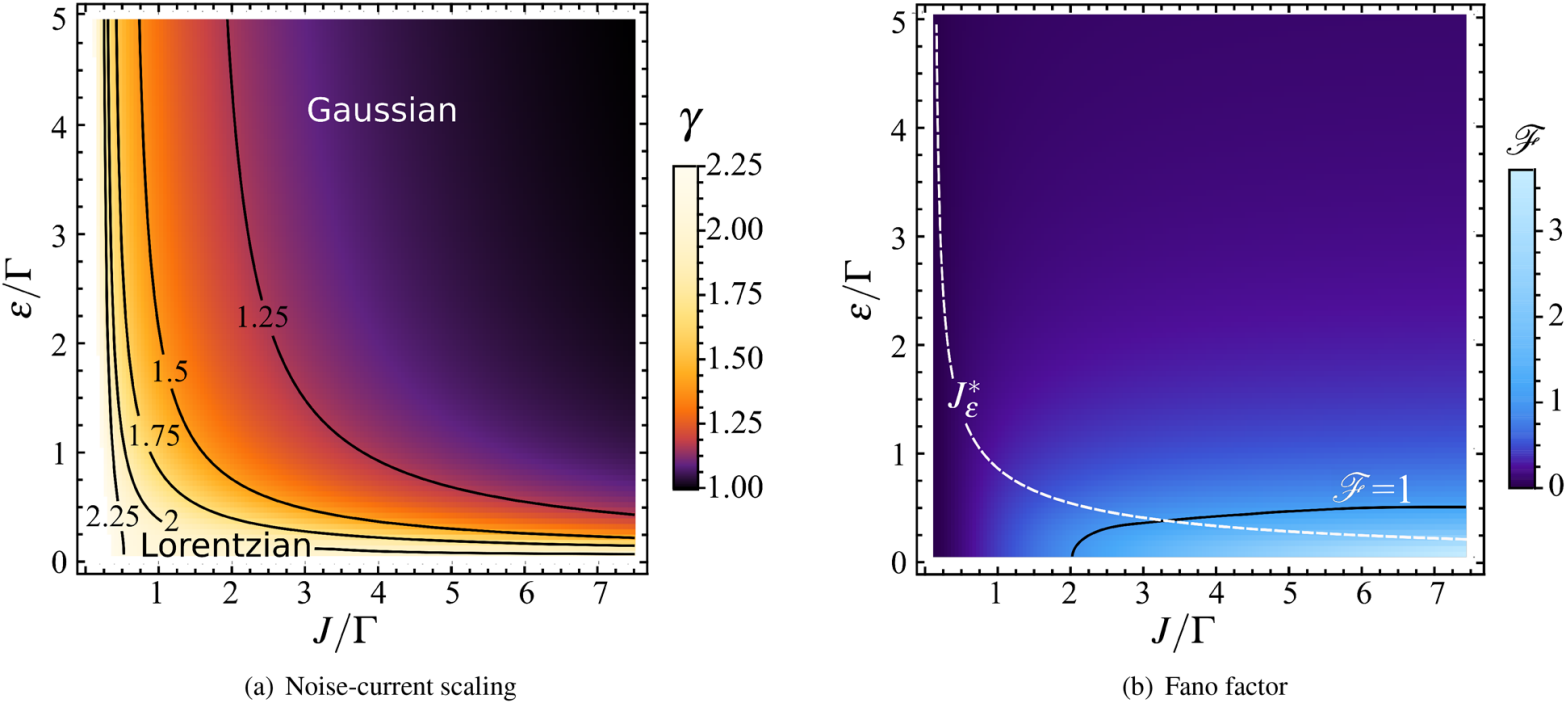
(**a**) Results for scaling exponent $\gamma$ in noise-current relation $S\propto J^\gamma$ as functions of the photon current *J* and anharmonicity $\varepsilon$. Color map represents logarithmic derivative $\gamma =\frac{d \ln S}{d\ln J}$. Contours of constant $\gamma$ show a crossover between Lorentzian and Gaussian pseudothermal light. The Lorentzian light corresponds to single-particle transport with Breit-Wigner transmission and $\gamma =2$, and the Gaussian is to many-body transport and the shot noise. The fractional $\gamma =3/2$ asymptotic appears at low currents and large anharmonicity $\varepsilon /\Gamma >1$. (**b**) Color map for Fano factor ${\mathcal{F}}= S/J$. The white dashed curve is $J^*_\varepsilon$. The black solid line stands for ${\mathcal{F}}=1$ contour.

This scaling resembles a situation of equilibrium radiation in a cavity at a very high temperature where the variation, $\langle \!\langle n^2\rangle \!\rangle =\langle n^2\rangle - \langle n\rangle ^2$, and average photon number are related as $\langle \!\langle n^2\rangle \!\rangle \sim \langle n\rangle ^2$. The upper boundary for the current $J^*_\varepsilon$ can be large or small compared to $\Gamma$ depending on the ratio $\varepsilon /\Gamma$. As shown in Fig. 4(a), the region of small $\varepsilon$ corresponds to a Lorentzian light with the exponent $\gamma \approx 2$ in the noise-current relation represented as $S\propto J^\gamma$. We note that the quadratic dependence and the Lorentzian spectrum of $S_{\mathrm{therm}}$ at finite $\omega$ (see Section “Generating functional method” and Eq. (39) for details), is analogous to a modulation noise in quantum point contacts^45^.

In the many-body regime with Gaussian light the following expression is found

8 $$\begin{aligned} S=\frac{\sqrt{\pi }}{2\sqrt{2}}\frac{J\Gamma }{\varepsilon \sqrt{1+\Gamma /J}} \ , \quad J\gg J^*_\varepsilon \ . \end{aligned}$$

The absence of the quadratic scaling in *S*(*J*) indicates the shot noise behavior. Also, this means that the Gaussian pseudothermal light has a partial coherence. In the limit of strong anharmonicity, $\varepsilon \gg \Gamma$, the scale $J^*_\varepsilon \sim \frac{\Gamma ^3}{\varepsilon ^2}$ is small compared to $\Gamma$. Consequently, the ratio $\Gamma /J$ in the square root of (8) can be both large or small compared to unity. This shows that two different asymptotical regimes of the Gaussian noise do exist, they are associated with $\Gamma /J\rightarrow 0$ and $\Gamma /J\rightarrow \infty$ limits. These limits have a continuous crossover between each other at $\Gamma /J\sim 1$.

If the drive is sufficiently strong, such that $J\gg \Gamma$ holds, then we arrive at the linear shot noise-current relation $S_{\mathrm{shot}} =\frac{\sqrt{\pi }}{2\sqrt{2}}\frac{\Gamma }{\varepsilon }J$. We note that the scalings similar to $S_{\mathrm{shot}}\propto J$ and $S_{\mathrm{therm}}\propto J^2$ were discussed in Refs.^46,47^. Namely, the photon current emitted by a coherent source and propagated through a random media shows a linear noise-current relation if the scattering region is absorbing, and quadratic if it is amplifying.

**Figure 5:**
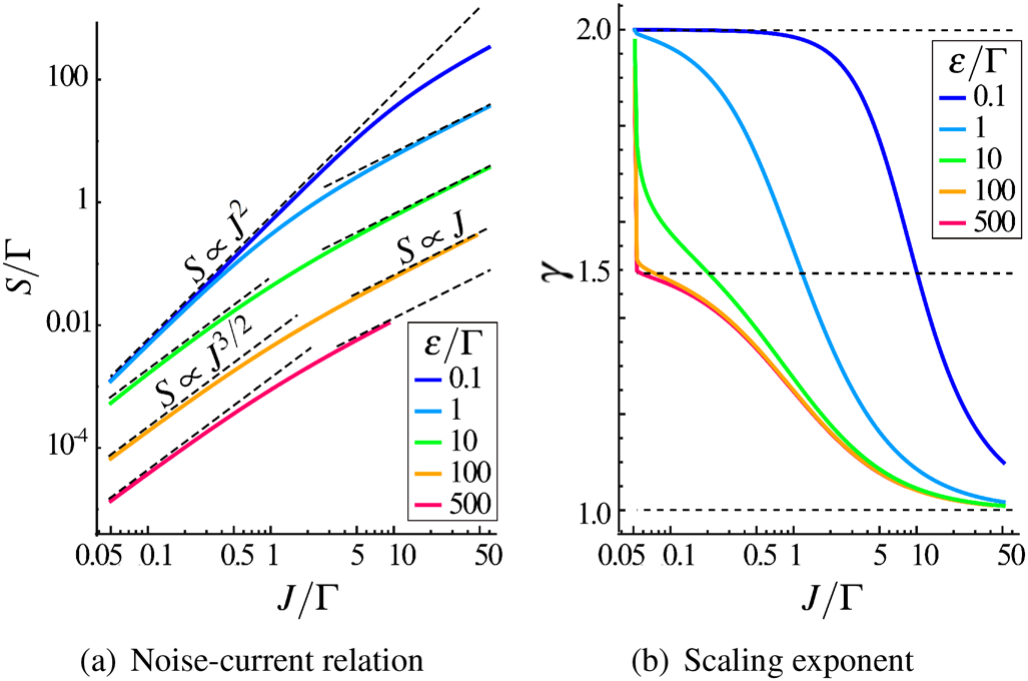
(**a**) Noise-current relation $S\propto J^\gamma$ for weak ($\varepsilon /\Gamma =$0.1), intermediate ($\varepsilon /\Gamma =$1) and strong ($\varepsilon /\Gamma =$10, 100, 500) anharmonicity (Bose-Hubbard interaction strength). The logarithmic scale demonstrates three different regimes of the photon transport: quadratic thermal behavior at weak interaction, and fractional power-law $S\propto J^{3/2}$ at small currents that changes to conventional shot noise $S\propto J$ as *J* increases. (**b**) Scaling exponent $\gamma$ that demonstrates a change of fixed point from $\gamma =2$ to $\gamma =3/2$ as the interaction increases.

At low currents, $\Gamma \gg J \gg J^*_\varepsilon$, the ratio $\Gamma /J$ in (8) dominates over the unity and we arrive at unconventional scaling of the noise, $S_{\mathrm{shot}}'\propto J^{3/2}$. This is one of the remarkable findings of this work. The change of the scaling exponent in $S\propto J^\gamma$ from $\gamma =2$ to $\gamma =3/2$ as $\varepsilon$ increases can be understood as emerging of a partial coherence in the propagated light. The difference between these nonequilibrium fixed points is illustrated by blue and orange curves in Figs. 5(a) and 5(b). In the limit of weak anharmonicity, $\varepsilon \ll \Gamma$, the asymptotic behavior of $S\propto J^{3/2}$ does not appear. Here, we obtain a simple crossover between the thermal-like behavior and the shot noise. It is demonstrated as blue curves, which start at $\gamma =2$ and saturate smoothly at $\gamma =1$. Parameter domains corresponding to different asymptotic limits are collected in Table 1.

### Fano factor

It is instructive to analyze the Fano factor, the ratio of the low-frequency noise and current, ${\mathcal{F}}=S/J$. In mesoscopic physics, ${\mathcal{F}}$ defines an effective charge that is transmitted coherently. This applies, e.g., to single-electron transistors^48^, to helical electrons scattering^49,50^ and to multiple Andreev reflection in superconducting junctions^51^. In our optical system, it can be associated with the emergence of positive (${\mathcal{F}} >1$) and negative (${\mathcal{F}} <1$) correlations between photons^52,53^.

In the regime of conventional shot noise we find universal result ${\mathcal{F}}_{\mathrm{shot}}=\sqrt{\frac{\pi }{8}} \frac{\Gamma }{ \varepsilon } \ll 1$ that does not depend on the drive amplitude. The unconventional shot noise $S_{\mathrm{shot}}'\propto J^{3/2}$ corresponds to Fano factor ${\mathcal{F}}'_{\mathrm{shot}}=\sqrt{\frac{\pi }{8} \frac{\Gamma J}{ \varepsilon ^2}}$ which is also smaller than unity. The regime of Lorentzian light yields ${\mathcal{F}}_{\mathrm{therm}}= \frac{J}{2\Gamma }$ which can be less or greater than unity. Thus, the Fano factor represents the complexity of nonequilibrium properties of the emitted light. Its color plot, the contours with ${\mathcal{F}}=1$ (black curve), and $J^*_\varepsilon$ (white dashed curve) are presented in Fig. 4(b).

## Discussion and outlook

Studies of driven-dissipative optical systems with Bose-Hubbard interaction is a large research area. In particular, incoherent drive effects were explored in experiments on the photon blockade^17,18^ and thermal states propagation^24^. The photon blockade is characterized by the antibunching of photons and their sub-Poissonian statistics. The thermal states propagation is an alternative regime, which is characterized by a suppressed photon blockade. In that experiment^24^, the bunching effect and super-Poissonian statistics of emitted photons were observed. In this work, we investigated how the incoherent pump influences the photon transport in a wideband limit and arbitrary excitations number. Namely, the central question of this work is how does a photonic counterpart of thermal transport through an electronic quantum dot with a non-equidistant spectrum look like? This question seems actual in view of increasing interest to novel phenomena and phases in nonequilibrium cavity and circuit QED where the fluctuation-dissipation relation and detailed balance condition are violated.

The bandwidth of the drive signal is assumed to be greater than characteristic relaxation rates. In this limit, we found that the increase of the Bose-Hubbard interaction and incoherent pump induce an intriguing transition from effectively thermal fluctuations to partially coherent nonequilibrium noise. A complexity of this transition is demonstrated in Fig. 5(b) where cross-sections of the phase diagram from Fig. 4(a) are shown for particular values of the interaction.

First, the Lorentzian transmission function and noise spectrum are transformed into Gaussian. The latter is a signature of many-body interaction in the anharmonic cavity and the consequence of transmitted photons decoherence. The time-resolved intensity correlator $g^{(2)}$ and photons dephasing reveal transitions from exponential to Gaussian decay laws demonstrating a certain similarity with Kubo model of an oscillator with stochastic frequency and also with the spin-boson model with sub-Ohmic $1/\omega$ spectrum.

Second, the low-frequency noise *S* and the Fano factor are nonlinear functions of the drive amplitude, or, the average transmitted current. The scaling exponent in the noise-current ratio, $S\propto J^{\gamma }$, can have three universal values. They indicate (i) single-photon transfer and effectively thermal state noise with $\gamma =2$, and (ii) partial coherence in strongly nonequilibrium limit with the shot noise ($\gamma =1$) and suppression of the Fano factor that becomes smaller than unity, and (iii) unconventional noise-current relation with the fractional exponent $\gamma =3/2$. In the regime (iii), that is found for low currents, the fluctuations of outgoing states are contributed by two competing components, the coherent emission and pseudothermal dissipative dynamics in a large Fock space of the cavity mode. This regime is supposed to be relevant for experiments reported in the Ref.^24^, where the difference between fluctuations in thermal radiation and the Poissonian regime was observed, and also in Refs.^21–23^, where the statistics of incident photons was measured by a continuous wave mixing.

In our solution, we found a single maximum in the transmission spectrum at the frequency $\omega _{\mathrm{ac}}$. This is in contrast to the case of the coherent drive at a particular frequency, where multi-photon supersplittings^54^ are clearly observed in experiments^14^. Their absence in our solution is explained by the averaging over a large amount of multi-photon transitions induced by the wideband and noisy drive.

The effective Caldeira–Leggett action proposed here describes fluctuations in the Gaussian (quadratic) order approximation. This approach provides a quasi-classical theory for noise represented as symmetrized correlators due to the Keldysh time ordering. The non-Gaussian fluctuations effects are the intriguing direction of recent studies related to non-linear quantum environment^35^ and dynamical Coulomb blockade^55^; in particular, the possibility of different time orderings on the Keldysh contour becomes important in the description of quantum noise and nonsymmetrized correlators.

## Summary

To conclude, we investigated steady-state photon transport in the anharmonic single-mode oscillator. It can be transmon qubit or nonlinear optical cavity incoherently driven and coupled to the input and output waveguides. We predict a rich behavior of the noise and intensity correlators in the nonequilibrium. The nonequilibrium conditions reveal intriguing effects due to the photon-photon interaction, they include pseudothermal fluctuations with partial coherence, Gaussian transmission spectrum with effectively sub-Ohmic dephasing, and unconventional shot noise with fractional power-law scaling.

The assumption of the wideband noisy drive field allows for a compact analytical solution. The decoherence due to the photon-photon interaction is described within a nonperturbative approach reduced to Caldeira-Leggett effective action. In this many-body problem, a stochastic phase of transmitted photons depends on various multi-photon transitions. We expect that the nontrivial behavior of the noise might not have a direct analogy in condensed matter systems.

The methodology is based on the Keldysh field theory. The solution for a system far from equilibrium is obtained. Our results give an insight into photon transport in the cavity and circuit QED, where the interaction and nonequilibrium dynamics appear on an equal footing. They can be generalized, in particular, for driven-dissipative Bose-Hubbard lattices^56,57^. It is suggested that these findings are experimentally accessible by methods based on photon counting^6,17^, homodyne detection of intensity correlations^16,18,24^, dispersive readout and spectroscopy^13,14^.

**Table 1:** Noise regimes classification

Interaction	Thermal noise, $S_{\mathrm{therm}}\propto J^2$. Lorentzian light	Fractional scaling, $S_{\mathrm{shot}}'\propto J^{3/2}$. Gaussian light	Conventional scaling, $S_{\mathrm{shot}}\propto J$. Gaussian light
weak, $\varepsilon \ll \Gamma$	$J\ll \frac{\Gamma ^2}{\varepsilon }$	–	$J\gg \frac{\Gamma ^2}{\varepsilon }$
strong, $\varepsilon \gg \Gamma$	$J\ll \frac{\Gamma ^3}{\varepsilon ^2}$	$\frac{\Gamma ^3}{\varepsilon ^2}\ll J\ll \Gamma$	$J \gg \Gamma$

## Methods

In this part of the paper, we present the methodology of calculations. We start from a formulation of nonequilibrium field theory that corresponds to a microscopic Hamiltonian (2). It is based on the Keldysh Green functions approach and Hubbard-Stratonovich transformation decoupling the non-Gaussian interaction term. In particular, the Landauer formula for photon current is derived within the Keldysh formalism. Then, the Caldeira-Leggett action is formulated and applied to calculations of decoherence, transmission function, noise spectrum, and $g^{(2)}$-correlator.

### Effective functional

#### Keldysh approach

The nonequilibrium field theory is started from the partition function given by the path integral, $Z=\int D[{{\bar{\Psi }}}, \Psi ]{\mathcal{T}} \exp (i{\mathbf {S}}[{{\bar{\Psi }}}, \Psi ])$, where ${\mathbf {S}}=\int _{{\mathcal{K}}} (i{{\bar{\Psi }}}\partial _t\Psi - H)dt$ is the action determined on Keldysh time contour ${\mathcal{K}}$. Here, ${\mathcal{T}}$ stands for Keldysh time ordering along ${\mathcal{K}}$. The vector with all path integral complex variables is $\Psi =[b(t,k),a(t),c(t,k)]^T$, and ${{\bar{\Psi }}}=[{{\bar{b}}}(t,k),\bar{a}(t),{{\bar{c}}}(t,k)]^T$ is its conjugate. Let us consider first the Keldysh action of the anharmonic cavity

9 $$\begin{aligned} i {\mathbf {S}}_{\mathrm{ac}}=i\int _{{\mathcal{K}}} \Big (\bar{a}(t)(i\partial _t-(\omega _0-\varepsilon ))a(t)- \varepsilon {{\bar{a}}}(t) a(t) {{\bar{a}}}(t) a(t) \Big )dt \ . \end{aligned}$$

By the virtue of Hubbard-Stratonovich real field $\phi$ we decouple the interaction term in (9) as follows

10 $$\begin{aligned} \exp \Big (-i\int _{{\mathcal{K}}} \varepsilon {{\bar{a}}}(t) a(t) {{\bar{a}}}(t) a(t) dt \Big ) = \int \!\! D [\phi ] \exp \Big (\int _{{\mathcal{K}}} \frac{i }{4\varepsilon }\phi ^2(t) dt - i\! \int _{{\mathcal{K}}} {{\bar{a}}}(t) \phi (t) a(t) dt \Big ) \ . \end{aligned}$$

The last term in the exponent corresponds to fluctuating potential $\phi (t)$ that randomly modulates oscillator frequency. As a result of this transformation, the cavity action becomes the following:

11 $$\begin{aligned} i{\mathbf {S}}_{\mathrm{ac}} \rightarrow \int _{{\mathcal{K}}} \frac{i }{4\varepsilon }\phi ^2(t) dt - i\! \int _{{\mathcal{K}}} \bar{a}(t)\big (i\partial _t-(\omega _0-\varepsilon +\phi (t) )\big )a(t) dt \ . \end{aligned}$$

With the use of this representation, the total action is formulated through matrix Green functions. Here, a transformation to the usual time axis $t\in [-\infty , \infty ]$ doubles the number of variables, $\Psi (t_{{\mathcal{K}}})\rightarrow [\Psi _+(t), \Psi _-(t)]$ and $\phi (t_{{\mathcal{K}}}) \rightarrow [\phi _+(t), \phi _-(t)]$, where these fields reside on upward or backward parts of the Keldysh contour labeled by ± indices. This is associated with Keldysh ${\check{\tau }}$-space parametrized by Pauli matrices ${{{\check{\tau }}}}_{x,y,z}$ and identity matrix ${{{\check{\tau }}}}_0$.

After the standard Keldysh rotation to classical and quantum components for complex fields, $\Psi _{c,q}(t)=\frac{1}{\sqrt{2}}\big (\Psi _+(t) \pm \Psi _-(t)\big )$, and for real fields, $\phi _{c,q}(t)=\frac{1}{2}\big (\phi _+(t) \pm \phi _-(t)\big )$, we arrive at the following representation of the total action:

12 $$\begin{aligned} i{\mathbf {S}}= \int \frac{i }{\varepsilon }\phi _c(t)\phi _q(t) dt + i \int {{\bar{\Psi }}} (t) \begin{bmatrix} {\check{G}}_{\mathrm{L}}^{-1} && -t_{\mathrm{L}}{{\check{\tau }}}_x && 0 \\ \\ -t_{\mathrm{L}}^*{{\check{\tau }}}_x && {\check{G}}_{\mathrm{ac}}^{-1}[\phi ] && -t_{\mathrm{R}}^*\tau _x \\ \\ 0 && - t_{\mathrm{R}}{{\check{\tau }}}_x && {\check{G}}_{\mathrm{R}}^{-1} \end{bmatrix} \Psi (t) dt \ , \quad \Psi (t)=\begin{bmatrix} \Psi _c(t) \\ \\ \Psi _q(t) \end{bmatrix} \ . \end{aligned}$$

Each element of the matrix is a block in ${{\check{\tau }}}$-space that possesses causality structure. Let us consider the left waveguide’s block:

13 $$\begin{aligned} {\check{G}}_{\mathrm{L}}^{-1} = \delta _{k,k'}\begin{bmatrix} 0 && i\partial _t-E_{\mathrm{L},k}- i0 \\ \\ i\partial _t-E_{\mathrm{L},k}+i0 && 2i 0 f_{\mathrm{L},k} \end{bmatrix} \ . \end{aligned}$$

The retarded (advanced) Green functions are $G^{R(A)}=1/(\omega -E_{\mathrm{L},k}\pm i0)$), they differ by the sign of the infinitesimal frequency shift *i*0 along the imaginary axis. The Keldysh component $2i 0 f_{\mathrm{L},k}$ encodes a distribution function $N_{\mathrm{L},k}$ of incident photons as $f_{\mathrm{L},k}=2N_{\mathrm{L},k}+1$. The similar definition applies for ${\check{G}}_{\mathrm{R}}^{-1}$. The local in time inverted Green function of the cavity

14 $$\begin{aligned} {\check{G}}_{\mathrm{ac}}^{-1}[\phi ] = (i\partial _t-\omega _0+\varepsilon -\phi _c(t) ){{\check{\tau }}}_x -\phi _q(t){{\check{\tau }}}_0 \end{aligned}$$

is nonstationary because it involves $\phi _c(t)$ and $\phi _q(t)$. The nondiagonal elements $t_{\mathrm{L,R}}{{\check{\tau }}}_x$ in (12) mix the cavity and waveguides’ fields corresponding to *k* and *p* modes. After the integration over $b_k$ and $c_p$, we obtain the effective action for the anharmonic cavity,

15 $$\begin{aligned} i{\mathbf {S}}_{\mathrm{ac}}[{{\bar{a}}},a,\phi ]=i\int {{\bar{a}}}(t){\check{{\mathscr{G}}}}_{\mathrm{ac}}^{-1}[\phi (t)]a(t) dt \ , \end{aligned}$$

where the inverted Green function

16 $$\begin{aligned} {\check{{\mathscr{G}}}}_{\mathrm{ac}}^{-1}[\phi ] = \delta (t - t') {\check{G}}_{\mathrm{ac}}^{-1} [\phi (t')]+ {{\check{\Sigma }}}(t-t') \end{aligned}$$

becomes a nonlocal in time due to the self-energy ${\check{\Sigma }}(t)=\int \frac{d\omega }{2\pi }e^{-i\omega t}{\check{\Sigma }}_\omega$. It has the causality structure, $${\check{\Sigma }}_\omega = \begin{bmatrix} 0 && \Sigma ^{A}_\omega \\ \Sigma ^{R}_\omega && \Sigma ^{K}_\omega \end{bmatrix}$$, the retarded and advanced components are $\Sigma ^{R(A)}_\omega =\pm i \Gamma$ where the total relaxation $\Gamma = \Gamma _{\mathrm{L}}+\Gamma _{\mathrm{R}}$ is a sum of the rates determined by the couplings to left and right waveguides, $\Gamma _{\mathrm{L,R}}=\pi \nu _{\mathrm{L,R}} |t_{\mathrm{L,R}}|^2$. The Keldysh component, $\Sigma ^{K}_\omega = 2 i \Gamma (2N_{\mathrm{ac},\omega }+1)$, brings information on the nonequilibrium distribution function in the cavity mode $N_{\mathrm{ac},\omega } = \frac{\Gamma _{\mathrm{L}}}{\Gamma }N_{\mathrm{L}, \omega } + \frac{\Gamma _{\mathrm{R}}}{\Gamma }N_{\mathrm{R}, \omega }$.

The integration over the cavity fields ${{\bar{a}}} , a$ provides the effective action for the fluctuating potential

17 $$\begin{aligned} i{\mathbf {S}}_{\mathrm{eff}}[\phi _c,\phi _q] = \int \frac{i}{ \varepsilon } \phi _c \phi _q dt-{\mathrm{Tr}} \ln \big ({\check{{\mathscr{G}}}}_{\mathrm{ac}}^{-1}[\phi _c,\phi _q]\big ) \ . \end{aligned}$$

The trace and logarithm here are determined over discretized time axis. As long as this action is non-Gaussian due to the matrix logarithm, we apply Gaussian expansion near a saddle point.

#### Saddle point equation

A configuration of $\phi _c(t)$ that determines a saddle point of ${\mathbf {S}}_{\mathrm{eff}}$ is given by the zero variation with respect to $\phi _q$, $\frac{\delta }{\delta \phi _q}{\mathbf {S}}_{\mathrm{eff}}[\phi _c,\phi _q]=0$. Applying this to (17), we find $\int \phi _c dt -i \varepsilon {\mathrm{Tr}} {\check{{\mathscr{G}}}}_{\mathrm{ac}} [\phi _c] =0$. Here, we use the standard notation for trace ${\mathrm{Tr}} (f(t-t'))=f(0) \int dt = \int \frac{d\omega }{2\pi } f_\omega \int dt$, where the time integral stands for a large constant that cancels out in the saddle point equation. In our system, the saddle point configuration is associated with the static part of the field, $\phi _0$. An explicit form of this equation is

18 $$\begin{aligned} \phi _0 - \varepsilon -\frac{\varepsilon \Gamma }{\pi } \int \frac{N_{\mathrm{ac},\omega } d\omega }{(\omega _0-\varepsilon +\phi _0)^2+\Gamma ^2} =0 \ . \end{aligned}$$

In our case of flat $N_{\mathrm{L},\omega }= F$ in the range $\omega \in [\omega _0-\Delta ; \ \omega _0+\Delta ]$ with $\Delta \gg \Gamma , \varepsilon$, and the absence of incident modes in the right waveguide, *i.e.*, $N_{\mathrm{R},\omega }=0$, the integral in (18) gives a constant that does not depend on $\phi _0$. The equation (18) becomes linear and we find $\phi _0= \varepsilon +F\varepsilon \frac{\Gamma _{\mathrm{L}}}{\Gamma }$.

Thus, the photon Green function at the saddle point is found after the inversion of ${\check{{\mathscr{G}}}}_{\mathrm{ac}}^{-1}[\phi ]$ from (16) with $\phi =\phi _0$:

19 $$\begin{aligned} {\check{{\mathscr{G}}}}_{\mathrm{ac},\omega }[\phi _0] = \begin{bmatrix} {\mathscr{G}}_{\mathrm{ac},\omega }^K && {\mathscr{G}}_{\mathrm{ac},\omega }^R \\ \\ {\mathscr{G}}_{\mathrm{ac},\omega }^A && 0 \end{bmatrix}\ , \quad {\mathscr{G}}_{\mathrm{ac},\omega }^{R(A)}=\frac{1}{\omega - (\omega _0-\varepsilon +\phi _0)\pm i\Gamma } \ , \quad {\mathscr{G}}_{\mathrm{ac},\omega }^K =\frac{-2i\Gamma (N_{\mathrm{ac},\omega }+1)}{(\omega -(\omega _0-\varepsilon +\phi _0) )^2+\Gamma ^2} \ . \end{aligned}$$

#### Caldeira-Leggett approach for fluctuations

Non-stationary configurations of the field, $\delta \phi _c(t)=\phi _c(t)-\phi _0$, determine many-body transitions which induce decoherence. Let us go back to the effective action ${\mathbf {S}}_{\mathrm{eff}}$. It is non-Gaussian because of the matrix logarithm. We apply quasi-classical second-order expansion of the logarithm by stochastic, $\delta \phi _c$, and quantum, $\phi _q$, fluctuations:

20 $$\begin{aligned} {\mathrm{Tr}} \ln \big ({\check{{\mathscr{G}}}}_{\mathrm{ac}}^{-1}[\phi (t) ]\big ) = {\mathrm{Tr}} \ln \big ({\check{{\mathscr{G}}}}_{\mathrm{ac}}^{-1}[\phi _0 ]-\delta \phi _c {{\check{\tau }}}_x -\phi _q{{\check{\tau }}}_0\big ) \approx {\mathrm{Tr}} \ln \big (\check{{\mathscr{G}}}_{\mathrm{ac}}^{-1}[\phi _0 ]\big )-\frac{1}{2}{\mathrm{Tr}}\left({\check{{\mathscr{G}}}}_{\mathrm{ac}} [\phi _0 ] \big (\delta \phi _c {{\check{\tau }}}_x+ \phi _q{{\check{\tau }}}_0\big ) \right) ^2 \ . \end{aligned}$$

This approximation for ${\mathbf {S}}_{\mathrm{eff}}$ provides Caldeira-Leggett action (3) which rules fluctuational and dissipative dynamics of $\phi (t)$. For nonequilibrium distributions indicated above, we find from (20) that the retarded and advanced parts in (3) read

21 $$\begin{aligned} \alpha ^{R(A)}_\omega = \frac{\pm 16i}{\pi \Gamma \Delta ^3} \varepsilon F^2 \Gamma _{\mathrm{L}}^2 + O[\Delta ^{-4}]\ . \end{aligned}$$

They are not universal, *i.e.*, they depend on the bandwidth and vanish as $1/\Delta ^{ 3}$. The Keldysh part, however, is non-zero

22 $$\begin{aligned} \alpha ^K_\omega = 16 \frac{ \Gamma _{\mathrm{L}} F (\Gamma +\Gamma _{\mathrm{L}} F)}{\Gamma \left( 4 \Gamma ^2+\omega ^2\right) } \ . \end{aligned}$$

This fact demonstrates a break of fluctuation-dissipation relation out of the equilibrium, *i.e.*, $\alpha ^K_\omega \ne (2N_{\mathrm{ac},\omega }+1) \left( \alpha ^R_\omega - \alpha ^A_\omega \right)$.

We note that the action ${\mathbf {S}}_{\mathrm{CL}}$ determines stochastic Langevin equation $\left( \frac{1}{\varepsilon }+\frac{1}{2}\alpha ^{R}_\omega \right) \delta \phi _{c,\omega }=\xi _\omega$ where the stochastic force $\xi$ has the correlator given by $\langle \xi _{-\omega }\xi _\omega \rangle =\frac{1}{4}\alpha ^K_\omega$. Addressing the photons decoherence in Keldysh formalism, we introduce stochastic $\Phi (t)=\int \limits ^t_{-\infty }\delta \phi _c(t')dt'$ and quantum $\varphi (t)=\int \limits ^t_{-\infty } \phi _q(t')dt'$ phases. In our case with vanishing $\alpha ^R$, the Langevin equation for the transmitted photon phase is reduced to

23 $$\begin{aligned} \frac{d}{dt} \Phi (t)= \varepsilon \xi (t) \end{aligned}$$

that corresponds to a frictionless drift.

### Calculations of transmission functions

#### Non-interacting limit. Lorentzian spectrum. Analogy with Landauer formula

We address a photon transport in a spirit of the Landauer formula determined by the transmission probability $T_\omega$. We start from a single-particle problem at $\varepsilon =0$. This limit is exactly solvable. After that, we study the case of $\varepsilon \ne 0$ taking into account many-body interaction utilizing Caldeira-Leggett action (3).

Let us define the photon current as time derivative of the photon number operator ${{\hat{n}}}_{\mathrm{R}}= \sum _p {{\hat{c}}}_p^\dagger {{\hat{c}}}_p$ in the right waveguide as $J=\frac{d}{dt}\langle {{\hat{n}}}_{\mathrm{R}}\rangle =\frac{i}{\hbar }\langle [{{\hat{H}}}, {{\hat{n}}}_{\mathrm{R}}]\rangle$. We note, that tunnelling Hamiltonians can be represented as ${{\hat{H}}}_{\mathrm{tL}}=t_{\mathrm{L}} \hat{{B}} ^ \dagger {{\hat{a}}}+ t_{\mathrm{L}}^* {{\hat{a}}} ^ \dagger \hat{{B}}$ and ${{\hat{H}}}_{{\mathrm{tR}}}=t_{\mathrm{R}} {\hat{C}} ^ \dagger {{\hat{a}}}+t_{\mathrm{R}}^* {{\hat{a}}}^ \dagger {\hat{C}}$ where $\hat{{B}}=\sum _k {{\hat{b}}}_k$ and ${\hat{C}}=\sum _p {{\hat{c}}}_p$ are ’local’ operators of waveguide fields. In this representation, the photon current reads as

24 $$\begin{aligned} J= i t_{\mathrm{R}}^*\langle {{\hat{a}}}^\dagger {\hat{C}} \rangle - i t_{\mathrm{R}}\langle {\hat{C}}^\dagger {{\hat{a}}} \rangle \ . \end{aligned}$$

We employ Keldysh Green functions technique to calculate these averages. In the non-interacting case, the action reads

25 $$\begin{aligned} i{\mathbf {S}}^{(0)} = i\! \int \frac{d\omega }{2\pi } {{\bar{\psi }}}_\omega {\mathbb {G}}_\omega ^{-1} \psi _\omega \ , \quad {\mathbb {G}}_\omega ^{-1} =\begin{bmatrix} {\check{g}}_{\mathrm{L,\omega }}^{-1} && -t_{\mathrm{L}}{{\check{\tau }}}_x && 0 \\ \\ -t_{\mathrm{L}}^*{{\check{\tau }}}_x && {\check{G}}_{0,\omega }^{-1} && -t_{\mathrm{R}}^*\tau _x \\ \\ 0 && - t_{\mathrm{R}}{{\check{\tau }}}_x && {\check{g}}_{\mathrm{R,\omega }}^{-1} \end{bmatrix} \ , \quad {\check{g}}_{\mathrm{L,R,\omega }}^{-1} = \begin{bmatrix} 0 && -i\frac{1}{\pi \nu _{\mathrm{L,R}}} \\ \\ i\frac{1}{\pi \nu _{\mathrm{L,R}}} && \frac{2i}{\pi \nu _{\mathrm{L}}}(2N_{\mathrm{L,R},\omega }+1) \end{bmatrix} \ . \end{aligned}$$

The inverted Green functions of the cavity mode is $\check{G}_{0,\omega }^{-1}= (\omega -\omega _0) {{\check{\tau }}}^x$. Green functions of left and right waveguides are, respectively, ${\check{g}}_{\mathrm{L, \omega }}=- \sum _k{\check{G}}_{\mathrm{L},\omega ,k}$ and ${\check{g}}_{\mathrm{R,\omega }} = - \sum _p{\check{G}}_{\mathrm{R,\omega },p}$. They act in space of variables $\psi =[ {B}_\omega , \ a_\omega , \ {C}_\omega ]$. The summations over *k* and *p* are found after replacements $\sum _{k,p} \rightarrow \nu _{\mathrm{L,R}}\int dE$.

Considering single-particle action ${\mathbf {S}}^{(0)}$, distribution functions $N_{\mathrm{L,R},\omega }$ can be arbitrary. Inverting ${\mathbb {G}}_\omega ^{-1}$ from (25), we find cavity-to-right waveguide propagator

26 $$\begin{aligned} {\check{g}}_{0\mathrm R,\omega } = -2i\pi \nu _{\mathrm{R}}t^*_{\mathrm{R}} \begin{bmatrix} \frac{ (\omega - \omega _0)( 2N_{\mathrm{R,\omega }} +1) - 2i\Gamma _{\mathrm{L}} (N_{\mathrm{R,\omega }} -N_{\mathrm{L,\omega }}) }{ \Gamma ^2 +(\omega - \omega _0)^2} && \frac{ 1/2}{ \omega - \omega _0 +i \Gamma } \\ \\ \frac{ -1/2}{ \omega - \omega _0 -i \Gamma } && 0 \end{bmatrix} \ . \end{aligned}$$

We use these expressions in the current (24): $\langle \hat{C} ^\dagger {{\hat{a}}} \rangle =\int i g^{< }_{0\mathrm R, \omega } \frac{d\omega }{2\pi }$ and $\langle {{\hat{a}}}^\dagger {\hat{C}} \rangle =\langle {\hat{C}} ^\dagger {{\hat{a}}} \rangle ^*$ where the ’greater’ Green function $g^{< }_{0\mathrm R, \omega } = \frac{1}{2}\left( g^K_{0\mathrm R, \omega } -g^R_{0\mathrm R, \omega } +g^A_{0\mathrm R, \omega } \right)$ is introduced. As a result, we arrive at the Landauer formula for the photon current

27 $$\begin{aligned} J=\int \tau _{ \omega }(N_{\mathrm{L,\omega }} -N_{\mathrm{R,\omega }} ) \frac{d\omega }{2\pi } \ . \end{aligned}$$

The transmission probability has the Lorentzian form $\tau _{ \omega } = \frac{4\Gamma _{\mathrm{L}}\Gamma _{\mathrm{R}}}{(\omega - \omega _0)^2+\Gamma ^2 }$. It reproduces unitary transmission $\tau _{\omega {=} \omega _0}=1$ at the resonance and symmetric relaxation rates $\Gamma _{\mathrm{L}}=\Gamma _{\mathrm{R}}=\Gamma /2$, the well-known result of the input-output theory^58^.

#### Interacting case: Gauge transformation

In this part, we present a generalization on the many-body problem where $\varepsilon \ne 0$ addressing the situation of flat distribution functions $N_{\mathrm{L} ,\omega }=F$ and $N_{\mathrm{R},\omega }=0$. The action with stochastic potential becomes local in time due to flat distributions

28 $$\begin{aligned} i{\mathbf {S}} = i\! \int dt \begin{bmatrix} {\bar{B}}_t&{{\bar{a}}}_t&{\bar{C}}_t \end{bmatrix} \begin{bmatrix} {\check{g}}_{\mathrm{L}}^{-1} && -t_{\mathrm{L}}{{\check{\tau }}}_x && 0 \\ \\ -t_{\mathrm{L}}^*{{\check{\tau }}}_x && {\check{G}}_{\mathrm{ac}}^{-1}[\phi ] && -t_{\mathrm{R}}^*\tau _x \\ \\ 0 && - t_{\mathrm{R}}{{\check{\tau }}}_x && {\check{g}}_{\mathrm{R}}^{-1} \end{bmatrix} \begin{bmatrix} {B}_t \\ \\ a_t \\ \\ {C}_t \end{bmatrix} \ . \end{aligned}$$

In contrast to the action in the non-interacting limit ${\mathbf {S}}^{(0)}$, the Green function ${\check{G}}_0^{-1}$ in the interacting case is replaced with ${\check{G}}_{\mathrm{ac}}^{-1}[\phi ]=(i\partial _t -\omega _0+\varepsilon -\phi _0-\delta \phi _c(t)){{\check{\tau }}}_x- \delta \phi _q(t) {{\check{\tau }}}_0$. The locality in time gives an advantage in analytic calculations. Namely, it is possible to get rid of time-dependent $\delta \phi _c(t)$ and $\phi _q(t)$ by the gauge transformation. This transformation from $\psi = [B, \ a, \ C]^T$ to new fields, $\psi '= [B', \ a', \ C']^T$, is distinct on upward and backward parts of ${\mathcal{K}}$ and reads

29 $$\begin{aligned} \psi _\pm (t)= e^{-i \Phi (t)\mp i \varphi (t)} \psi '_\pm (t) \ . \end{aligned}$$

It provides a nonperturbative solution for photon decoherence. A dynamics of these new fields $\psi '$ is ruled by the action ${\mathbf {S}}^{(0)}$ found for $\varepsilon =0$. Hence, the solution for cavity-to-right waveguide propagator ${\check{g}}_{\mathrm{ac R}}$ at $\varepsilon \ne 0$ is obtained from non-interacting result $\check{g}_{\mathrm{0R}}$ from (26) when the gauge inversed to (29) is applied:

30 $$\begin{aligned} {\check{g}}_{\mathrm{ac R} }(t,t') = \Big (\cos \varphi (t)\cos \varphi (t')- i \sin (\varphi (t) + \varphi (t'))\Big ) e^{-i(\Phi (t)-\Phi (t'))}e^{i(\varepsilon -\phi _0)(t-t')} {\check{g}}_{0{\mathrm{R}}}(t-t') . \end{aligned}$$

This function is not stationary due to the fluctuating potential. After the averaging ${\check{g}}_{\mathrm{ac R} }(t,t')$ with respect to ${\mathbf {S}}_{\mathrm{CL}}$, we arrive at the stationary Green function that depends on the time difference

31 $$\begin{aligned} \langle {\check{g}}_{\mathrm{ac R} }(t, t') \rangle = z(t-t') e^{-i\langle \Phi (t)\varphi (t')\rangle +i\langle \varphi (t)\Phi (t')\rangle } {\check{g}}_{\mathrm{0R}}(t-t') \ . \end{aligned}$$

The relevant modification appears in the envelope related to stochastic fluctuations $z(t)=\langle e^{-i(\Phi (t)-\Phi (0))}\rangle$. The average with Gaussian action ${\mathbf {S}}_{\mathrm{CL}}$ yields $z(t)=\exp (-D(t))$ where the symmetrized autocorrelation function is $D(t )=\frac{1}{2} (\langle \Phi (t) \Phi ( 0)\rangle + \langle \Phi (0) \Phi ( t)\rangle ) - \langle \Phi ^2(0)\rangle$. With the use of Langevin equation (23), we find that $D(t)=\frac{1}{4}\int ^t_0\int ^{t'}_0\alpha ^K(t'')dt''dt'$. The integration with Keldysh component $\alpha ^K (t)= F(F+2) e^{-2 \Gamma |t|}$, found after the Fourier transform of (22), gives

32 $$\begin{aligned} D(t)= \frac{ \Gamma _{\mathrm{L}} \varepsilon ^2 }{2 \Gamma ^3}\left( F +\frac{\Gamma _{\mathrm{L}}}{\Gamma } F^2 \right) \left( 2 \Gamma |t| +e^{-2 \Gamma |t|}-1\right) \ . \end{aligned}$$

Combinations of classical-quantum retarded and advanced correlators in (31) are due to the mixing of fields on different parts of the contour ${\mathcal{K}}$; they read $\langle \Phi (t)\varphi (0)\rangle =i\varepsilon t \theta (t)$ and $\langle \varphi (t)\Phi (0)\rangle =-i\varepsilon t \theta (-t)$. In our Gaussian theory, classical-quantum terms yield simply a complex phase $e^{-i\varepsilon (t-t')}$ that results in a shift of the cavity mode frequency

33 $$\begin{aligned} \omega _{\mathrm{ac}}= \omega _0 -\varepsilon +F\varepsilon \frac{\Gamma _{\mathrm{L}}}{\Gamma } \ , \end{aligned}$$

a quantum counterpart of the nonlinear frequency shift in a classical anharmonic oscillator.

#### Gaussian spectrum

The result (31) provides the expression for the transmission function in the time domain

34 $$\begin{aligned} T(t)=z(t) \tau (t) \ , \quad \tau (t) =2 \frac{\Gamma _{\mathrm{L}} \Gamma _{\mathrm{R}}}{ \Gamma } e^{-\Gamma |t| - i\omega _{\mathrm{ac}} t} \ . \end{aligned}$$

Here, $\tau (t)$ is Fourier transformed Lorentzian transmission with the maximum at $\omega _{\mathrm{ac}}$. The Fourier transform of (34) reads $T_\omega =\int z_{\omega -\omega '} \tau _{\omega '} \frac{d \omega '}{2\pi }$. Analytic calculation of this integral is challenging. Nevertheless, we find asymptotic expressions for the strongly nonequilibrium regime with the use of the saddle point method. In this solution, the correlator in the exponent of *z*(*t*) is approximated as $D(t)\approx \kappa ^2 t^2$. Here, new relaxation rate $\kappa = \varepsilon \sqrt{ \frac{\Gamma _{\mathrm{L}} }{\Gamma } \left( F+\frac{\Gamma _{\mathrm{L}}}{\Gamma } F^2\right) }$ appears. It shows that the coherence loss rate grows non-linearly as *F* increases. The Fourier transformation gives the transmission spectrum of Gaussian shape: $T_{\omega } = 2\frac{\sqrt{\pi }\Gamma _{\mathrm{L}}\Gamma _{\mathrm{R}}}{ \Gamma \kappa } \exp \left( \!-\frac{(\omega -\omega _{\mathrm{ac}})^2}{4\kappa ^2} \right)$. This result applies to a regime of strong driving or strong interaction, such that the condition $\kappa \gg \Gamma$ is satisfied. Let us analyze this condition for the symmetric case of $\Gamma _{\mathrm{L}}=\Gamma _{\mathrm{R}}= \Gamma /2$. The decay rate can be equivalently expressed through the current as $\kappa = \frac{\varepsilon }{\Gamma }\sqrt{J\Gamma +J^2}$ where $J=F\Gamma /2$ does not depend on anharmonicity in our problem with the wide spectrum of the incoherent drive. The condition $\kappa \gg \Gamma$ is resolved as $J\gg J^*_\varepsilon$:

35 $$\begin{aligned} J^*_\varepsilon =\frac{\Gamma }{2}\Big (\sqrt{1+4\frac{\Gamma ^2}{\varepsilon ^2}}-1 \Big )\approx {\left\{ \begin{array}{ll} \frac{\Gamma ^2}{\varepsilon } \ , & \varepsilon \ll \Gamma \ ; \\ \\ \frac{\Gamma ^3}{\varepsilon ^2} \ , & \varepsilon \gg \Gamma \ . \\ \end{array}\right. } \end{aligned}$$

The scale $J^*_\varepsilon$ corresponds to the lower boundary for the current at a given $\varepsilon$ when it drives the system out of the equilibrium and induces Gaussian chaotic light emission. If the current is small, $J\ll J^*_\varepsilon$, then the system is in a fixed point related to thermal behavior, as shown in the phase diagram in Fig. 4(a). We learn from (35) that there is two asymptotics in $J^*_\varepsilon$ that distinguish weak, $\varepsilon \ll \Gamma$, and strong, $\varepsilon \gg \Gamma$, interaction limits. Both of them overlap with the Lorentzian and Gaussian sectors.

#### Photon current and cavity photon number

As it follows from (27), the photon current at $N_{\mathrm{L}, \omega }=F$ and $N_{\mathrm{R}, \omega }=0$ reads

36 $$\begin{aligned} J=2 F \frac{\Gamma _{\mathrm{L}}\Gamma _{\mathrm{R}}}{\Gamma } \ . \end{aligned}$$

The average photon number in this limit,

37 $$\begin{aligned} \langle {{\hat{a}}}^\dagger {{\hat{a}}}\rangle =F \frac{ \Gamma _{\mathrm{L}} }{ \Gamma } \ , \end{aligned}$$

is obtained as $\langle {{\hat{a}}}^\dagger {{\hat{a}}}\rangle =\int i{\mathscr{G}}_{\mathrm{ac},\omega }^< \frac{d\omega }{2\pi }$ where the ’greater’ Green function is ${\mathscr{G}}_{\mathrm{ac},\omega }^< = \frac{1}{2}({\mathscr{G}}_{\mathrm{ac},\omega }^K -{\mathscr{G}}_{\mathrm{ac},\omega }^R +{\mathscr{G}}_{\mathrm{ac},\omega }^A )$. We note that the frequency shift due to the anharmonicity can be represented via (37) as $\omega _{\mathrm{ac}}=\omega _0 - \varepsilon +\langle {{\hat{a}}}^\dagger {{\hat{a}}}\rangle \varepsilon$. As it should be, the same value can be found from a mean-field approach for Bose-Hubbard interaction where ${{\hat{a}}}^\dagger {{\hat{a}}} {{\hat{a}}}^\dagger {{\hat{a}}} \rightarrow {{\hat{a}}}^\dagger {{\hat{a}}} \langle {{\hat{a}}}^\dagger {{\hat{a}}}\rangle$.

The current given by (36) is not modified by the interaction in the case of flat distribution functions. As follows from the representation of the current through $\langle g_{\mathrm{ac R} }^<(t, t) \rangle$ at coincident times, we find $J=F z(0) \int \frac{d\omega }{2\pi }\tau _{\omega }$ from (34). As long as $z(0)=1$ for any interaction, *J* does not depend on $\varepsilon$. The same logic applies to $\langle {{\hat{a}}}^\dagger {{\hat{a}}}\rangle$ which also does not change as $\varepsilon$ increases.

### Calculations of the noise and intensity fluctuations

#### Generating functional method

We start calculations of noise from the non-interacting limit and then generalize for the interacting case. Cumulants of transmitted photons can be calculated through variations of the generating functional logarithm, $\ln Z[\eta ]$. The generating functional is given by the path integral

38 $$\begin{aligned} Z[\eta ]=\int D[{{\bar{\psi }}}, \psi ]{\mathcal{T}}\exp (i{\mathbf {S}}[{{\bar{\psi }}}, \psi ]+i \eta {{\bar{\psi }}} {\mathbb {J}} \psi ) \ , \quad {\mathbb {J}} = \begin{bmatrix} 0 & 0 & 0 & 0 & 0 & 0 \\ 0 & 0 & 0 & 0 & 0 & 0 \\ 0 & 0 & 0 & 0 & 0 & 0 \\ 0 & 0 & 0 & 0 & i t_{\mathrm{R}}^* & 0 \\ 0 & 0 & 0 & 0 & 0 & 0 \\ 0 & 0 & -it_{\mathrm{R}} & 0 & 0 & 0 \\ \end{bmatrix} \ , \quad \psi (t) =\begin{bmatrix} B_+(t) \\ B_-(t) \\ a_+(t) \\ a_-(t)\\ C_+(t) \\ C_- (t)\\ \end{bmatrix} \ . \end{aligned}$$

Here, $\eta$ is generating variable and ${\mathbb {J}}$ parametrizes current from (24) through fields *a* and *C* on opposite branches (labeled by $\tau _z=\pm 1$) of the contour ${\mathcal{K}}$ as $J={{\bar{\psi }}} {\mathbb {J}} \psi$. The integration over ${{\bar{\psi }}}, \psi$ in (38) results in $\ln Z[\eta ]={\mathrm{Tr}}\ln (1+{\mathbb {G}}\eta {\mathbb {J}})$. Photon current *J* is found as first variation of $\ln Z[\eta ]$ by $\eta$, where it is set to zero $\eta \rightarrow 0$. This gives $J=\int \frac{d\omega }{2\pi }i {\mathrm{tr}}\big ( {\mathbb {G}}_\omega {\mathbb {J}}\big )$ that is reduced to Landauer formula (27); here, “${{\mathrm{tr}}}$” stands for trace over ${{\check{\tau }}}$, *L*, *R*, and oscillator indices. In order to find the spectrum of the symmetrized noise, $S ^{(0)}_\omega =\frac{1}{2} (\langle \delta J_{-\omega } \delta J_{\omega } \rangle +\langle \delta J_{\omega } \delta J_{-\omega } \rangle )$, we need to find second variation of $\ln Z[\eta ]$ by $\eta _{-\omega }$ and $\eta _{\omega }$. In our particular case of flat $N_{\mathrm{L},\omega }$, the spectrum is Lorentzian

39 $$\begin{aligned} S ^{(0)}_\omega = -\frac{1}{2}\int \frac{d\omega _1}{2\pi }i {\mathrm{tr}}\big ( {\mathbb {G}}_{\omega _1}{\mathbb {J}} {\mathbb {G}}_{\omega +\omega _1}{\mathbb {J}}\big ) =8F^2\frac{\Gamma _{\mathrm{L}}^2\Gamma _{\mathrm{R}}^2}{\Gamma (\omega ^2+4\Gamma ^2)} \ . \end{aligned}$$

#### Asymptotic behavior of the noise-current relation

Hereafter, we express *F* through the current *J* according to (36) and suppose that $\Gamma _{\mathrm{L}}$ and $\Gamma _{\mathrm{R}}$ can be unequal. This allows analyzing zero-frequency noise-current ratio, *S*(*J*), where both *S* and *J* are measured by the output detector. The zero-frequency noise found in (39) has quadratic scaling, $S_{\mathrm{therm}} = \frac{J^2}{2\Gamma }$ at $J\ll J^*_\varepsilon$ , that corresponds to Lorentzian light emitted into the output waveguide.

The decoherence is important at $J\gg J^*_\varepsilon$ when pseudothermal output light becomes Gaussian and the thermal noise is changed to shot noise. Technically, the inclusion of the stochastic $\Phi (t)$ in the noise calculation is performed with the gauge transformation, similarly to that applied for the transmission function *T*(*t*). Symmetrized correlator of the noise, $S(t)=\frac{1}{2}(\langle \delta J(t)\delta J(0)\rangle + \langle \delta J(0)\delta J(t)\rangle )$, reads $S(t)= z^2(t) S^{(0)}(t)$ where $S^{(0)}(t) = \frac{1}{2} J^2e^{-2\Gamma |t|}$ is time-resolved Lorentzian correlator found in the non-interacting limit (39). The shot noise expression follows from the Fourier transform of *S*(*t*) where the correlator in the exponent of *z*(*t*) is Gaussian $D(t)\approx \kappa ^2 t^2$. The low-frequency result, $S\equiv S_{\omega =0}$, is given by $S=J^2\int \limits _0^\infty e^{-2\Gamma |t|-2\kappa ^2 t^2}$. Assuming $\kappa \gg \Gamma$, that is equivalent to $J\gg J^*_\varepsilon$, we calculate this integral and arrive at one of central results

40 $$\begin{aligned} S = \sqrt{\frac{\pi }{2}} \frac{\Gamma _{\mathrm{R}}}{ \varepsilon } \frac{J}{\sqrt{1+2\Gamma _{\mathrm{R}}/J}}\ . \end{aligned}$$

Let us analyze it in details. If the interaction is weak, $\varepsilon \ll \Gamma$, then we always have $\Gamma _{\mathrm{R}}/J\ll 1$ according to $J^*_\varepsilon \sim \frac{\Gamma ^2}{\varepsilon }$ from (35). Thus, in the nonequilibrium regime at weak anharmonicity, we find conventional shot noise from (40) with a linear noise-current ratio $S_{\mathrm{shot}} = \sqrt{\frac{\pi }{2}} \frac{\Gamma _{\mathrm{R}}}{ \varepsilon } J$ at $J\gg \frac{\Gamma ^2}{\varepsilon }$. This result is considered as a nonequilibrium fixed point with the exponent $\gamma =1$.

For strong interaction, $\varepsilon \gg \Gamma _{\mathrm{L,R}}$, the thermal noise sector in Fig. 4(a) shrinks because of the vanishing $J^*_\varepsilon \sim \frac{\Gamma ^3}{\varepsilon ^2}$. This means that we can go to low current domain where $J\ll \Gamma _{\mathrm{R}}$. Here, the noise-current relation (40) has the following asymptotic

41 $$\begin{aligned} S_{\mathrm{shot}}' = \frac{\sqrt{\pi \Gamma _{\mathrm{R}}}}{2\varepsilon } J ^{3/2} \ , \quad \Gamma \gg J\gg \frac{\Gamma ^3}{\varepsilon ^2}\ . \end{aligned}$$

This non-analytical dependence occurs at small $J\sim J^*_\varepsilon$, *i.e.*, it is parametrically close to zero current line $J=0$ in Fig. 4(a) at large $\varepsilon$. This feature is also demonstrated in Fig. 5(b) for $\varepsilon >\Gamma$ where orange and red curves drop rapidly from $\gamma =2$ to $\gamma =3/2$. Then, curves saturate to conventional shot noise with $\gamma =1$ as the current increases.

There are smooth crossovers between $S_{\mathrm{therm}}$, $S_{\mathrm{shot}}'$ and $S_{\mathrm{shot}}$ at intermediate $\varepsilon \sim \Gamma$ as a consequence of fluctuations in zero-dimensional cavity. At weak anharmonicity, there is no $S_{\mathrm{shot}}'$ behavior. Instead, there is a crossover from $S_{\mathrm{therm}}$ to $S_{\mathrm{shot}}$ at $J \sim \frac{\Gamma ^2}{\varepsilon }$. It is shown in Fig. 5(b) where blue curve decays smoothly from $\gamma =2$ to $\gamma =1$.

#### Intensity correlators

Time-resolved intensity-intensity correlator in the right waveguide,

42 $$\begin{aligned} g^{(2)}(t)=\frac{\langle {{\hat{C}}}^\dagger \! (t) {{\hat{C}}}(t) {{\hat{C}}}^\dagger \! (0) {{\hat{C}}}(0)\rangle }{|\langle {{\hat{C}}}^\dagger \! (0) {{\hat{C}}}(0)\rangle |^2} \ , \end{aligned}$$

is an indicator for bunching or antibunching of photons in the output field. In our solution we assumed that anomalous term $\langle {{\hat{C}}}(t) {{\hat{C}}}(0)\rangle$ is zero, and this four-point correlator is defined through the two-point one, $g^{(2)}(t)=1+|g^{(1)}(t)|^2$, according to the Wick theorem applicable in our quasi-classical solution. We find $g^{(1)}(t)=z(t){\check{g}}_{\mathrm{RR}}(t )/{\check{g}}_{\mathrm{RR}}(0 )$ where ${\check{g}}_{\mathrm{RR}}(t )\propto e^{-\Gamma t}$ is the Lorentzian propagator while *z*(*t*) involves $\varepsilon$ and *F*.

Time dependence of the correlator shows exponential decay in the non-interacting limit $g^{(2)}(t)=1+e^{-2\Gamma t}$ at $t>0$. In the interacting case, we find

43 $$\begin{aligned} g^{(2)}(t)=1+ \mathrm{exp}\Big [- \frac{\kappa ^2}{\Gamma ^2}(2\Gamma t + e^{-2 \Gamma |t|}-1 )-2\Gamma t\Big ] \ . \end{aligned}$$

There is Gaussian law of the correlations decay $g^{(2)}(t)=1+e^{-2 \kappa ^2 t^2}$ for $t\ll 1/\Gamma$. For large timescale, $t\gg 1/\Gamma$, there is exponential decay $g^{(2)}(t)=1+e^{-2\frac{\kappa ^2}{\Gamma } t}$. As follows from the condition $\kappa \gg \Gamma$ on the nonequilibrium, the decay rate in the latter case exceeds that from the non-interacting limit as $\frac{\kappa ^2}{\Gamma } \gg \Gamma$.

## References

[1] Haroche S, Brune M, Raimond JM (2020). From cavity to circuit quantum electrodynamics. Nat. Phys..

[2] Blais, A., Grimsmo, A. L., Girvin, S. & Wallraff, A. Circuit quantum electrodynamics. *preprint* arXiv:2005.12667 (2020).

[3] Frisk Kockum, A., Miranowicz, A., De Liberato, S., Savasta, S. & Nori, F. Ultrastrong coupling between light and matter. *Nat. Rev. Phys.* **1**, 19–40 (2019).

[4] Kirton P, Roses MM, Keeling J, Dalla Torre EG (2019). Introduction to the Dicke model: From equilibrium to nonequilibrium, and vice versa. Adv. Quantum Technol..

[5] Miller R (2005). Trapped atoms in cavity QED: coupling quantized light and matter. J. Phys. B: At. Mol. Opt. Phys..

[6] Baumann, K., Guerlin, C., Brennecke, F. & Esslinger, T. Dicke quantum phase transition with a superfluid gas in an optical cavity. *Nature* **464**, 1301 EP – (2010). 10.1038/nature09009 20428162

[7] Safavi-Naini A (2018). Verification of a many-ion simulator of the Dicke model through slow quenches across a phase transition. Phys. Rev. Lett..

[8] Magazzù L (2018). Probing the strongly driven spin-boson model in a superconducting quantum circuit. Nat. Commun..

[9] Buchhold M, Strack P, Sachdev S, Diehl S (2013). Dicke-model quantum spin and photon glass in optical cavities: Nonequilibrium theory and experimental signatures. Phys. Rev. A.

[10] Clerk AA, Lehnert KW, Bertet P, Petta JR, Nakamura Y (2020). Hybrid quantum systems with circuit quantum electrodynamics. Nat. Phys..

[11] Kubo Y (2011). Hybrid quantum circuit with a superconducting qubit coupled to a spin ensemble. Phys. Rev. Lett..

[12] Srinivasan S, Hoffman A, Gambetta J, Houck A (2011). Tunable coupling in circuit quantum electrodynamics using a superconducting charge qubit with a $$v$$-shaped energy level diagram. Phys. Rev. Lett..

[13] Macha P (2014). Implementation of a quantum metamaterial using superconducting qubits. Nat. Commun..

[14] Braumüller J (2017). Analog quantum simulation of the rabi model in the ultra-strong coupling regime. Nat. Commun..

[15] Imamoglu, A., Schmidt, H., Woods, G. & Deutsch, M. Strongly interacting photons in a nonlinear cavity. *Phys. Rev. Lett.* **79**, 1467–1470 (1997).

[16] Birnbaum KM (2005). Photon blockade in an optical cavity with one trapped atom. Nature.

[17] Hoffman AJ (2011). Dispersive photon blockade in a superconducting circuit. Phys. Rev. Lett..

[18] Lang C (2011). Observation of resonant photon blockade at microwave frequencies using correlation function measurements. Phys. Rev. Lett..

[19] Jauho A-P, Wingreen NS, Meir Y (1994). Time-dependent transport in interacting and noninteracting resonant-tunneling systems. Phys. Rev. B.

[20] Giazotto F, Heikkilä TT, Luukanen A, Savin AM, Pekola JP (2006). Opportunities for mesoscopics in thermometry and refrigeration: Physics and applications. Rev. Mod. Phys..

[21] Dmitriev AY (2019). Probing photon statistics of coherent states by continuous wave mixing on a two-level system. Phys. Rev. A.

[22] Hönigl-Decrinis T, Shaikhaidarov R, de Graaf S, Antonov V, Astafiev O (2020). Two-level system as a quantum sensor for absolute calibration of power. Phys. Rev. Appl..

[23] Zhou Y, Peng Z, Horiuchi Y, Astafiev O, Tsai J (2020). Tunable microwave single-photon source based on transmon qubit with high efficiency. Phys. Rev. Appl..

[24] Goetz J (2017). Photon statistics of propagating thermal microwaves. Phys. Rev. Lett..

[25] Westig M (2017). Emission of nonclassical radiation by inelastic Cooper pair tunneling. Phys. Rev. Lett..

[26] Hofer PP, Brask JB, Perarnau-Llobet M, Brunner N (2017). Quantum thermal machine as a thermometer. Phys. Rev. Lett..

[27] Yao NY (2013). Quantum logic between remote quantum registers. Phys. Rev. A.

[28] Liew TCH, Savona V (2010). Single photons from coupled quantum modes. Phys. Rev. Lett..

[29] Bamba M, Imamoğlu A, Carusotto I, Ciuti C (2011). Origin of strong photon antibunching in weakly nonlinear photonic molecules. Phys. Rev. A.

[30] Kamenev, A. *Field theory of non-equilibrium systems* (Cambridge University Press, 2011).

[31] Altland, A. & Simons, B. D. *Condensed matter field theory* (Cambridge University Press, 2010).

[32] Caldeira AO, Leggett AJ (1981). Influence of dissipation on quantum tunneling in macroscopic systems. Phys. Rev. Lett..

[33] Shnirman A (2016). U(1) and SU(2) quantum dissipative systems: the Caldeira–Leggett versus Ambegaokar–Eckern–Schön approaches. J. Exp. Theor. Phys..

[34] Maghrebi MF, Gorshkov AV (2016). Nonequilibrium many-body steady states via Keldysh formalism. Phys. Rev. B.

[35] Wang Y-X, Clerk AA (2020). Spectral characterization of non-gaussian quantum noise: Keldysh approach and application to photon shot noise. Phys. Rev. Res..

[36] Dalla Torre EG, Diehl S, Lukin MD, Sachdev S, Strack P (2013). Keldysh approach for nonequilibrium phase transitions in quantum optics: Beyond the Dicke model in optical cavities. Phys. Rev. A.

[37] Sieberer LM, Huber SD, Altman E, Diehl S (2013). Dynamical critical phenomena in driven-dissipative systems. Phys. Rev. Lett..

[38] Gambetta J (2006). Qubit-photon interactions in a cavity: Measurement-induced dephasing and number splitting. Phys. Rev. A.

[39] Kubo, R. *A Stochastic Theory of Line Shape*, pp. 101–127 (Wiley, 2007).

[40] Eckern U, Schön G, Ambegaokar V (1984). Quantum dynamics of a superconducting tunnel junction. Phys. Rev. B.

[41] Shnirman A, Makhlin Y, Schön G (2002). Noise and decoherence in quantum two-level systems. Phys. Scripta.

[42] Gabelli J (2004). Hanbury Brown-Twiss correlations to probe the population statistics of GHz photons emitted by conductors. Phys. Rev. Lett..

[43] Zou XT, Mandel L (1990). Photon-antibunching and sub-poissonian photon statistics. Phys. Rev. A.

[44] Blanter Y, Büttiker M (2000). Shot noise in mesoscopic conductors. Phys. Rep..

[45] Kogan, S. *Electronic noise and fluctuations in solids* (Cambridge University Press, 2008).

[46] Beenakker C, Patra M (1999). Photon shot noise. Mod. Phys. Lett. B.

[47] Beenakker CWJ, Patra M, Brouwer PW (2000). Photonic excess noise and wave localization. Phys. Rev. A.

[48] Choi M-S, Plastina F, Fazio R (2003). Charge and current fluctuations in a superconducting single-electron transistor near a Cooper pair resonance. Phys. Rev. B.

[49] Nagaev KE, Remizov SV, Shapiro DS (2018). Noise in the helical edge channel anisotropically coupled to a local spin. JETP Lett..

[50] Kurilovich PD, Kurilovich VD, Burmistrov IS, Gefen Y, Goldstein M (2019). Unrestricted electron bunching at the helical edge. Phys. Rev. Lett..

[51] Cron R, Goffman MF, Esteve D, Urbina C (2001). Multiple-charge-quanta shot noise in superconducting atomic contacts. Phys. Rev. Lett..

[52] Shapiro DS, Pogosov WV, Lozovik YE (2020). Universal fluctuations and squeezing in a generalized Dicke model near the superradiant phase transition. Phys. Rev. A.

[53] Shapiro DS, Rubtsov AN, Remizov SV, Pogosov WV, Lozovik YE (2019). Fluctuations and photon statistics in a quantum metamaterial near a superradiant transition. Phys. Rev. A.

[54] Elliott M, Ginossar E (2016). Applications of the Fokker-Planck equation in circuit quantum electrodynamics. Phys. Rev. A.

[55] Zhan H, Rastelli G, Belzig W (2020). Non-classical current noise and light emission of an ac-driven tunnel junction. New J. Phys..

[56] Vicentini F, Minganti F, Rota R, Orso G, Ciuti C (2018). Critical slowing down in driven-dissipative Bose-Hubbard lattices. Phys. Rev. A.

[57] Biella A, Mazza L, Carusotto I, Rossini D, Fazio R (2015). Photon transport in a dissipative chain of nonlinear cavities. Phys. Rev. A.

[58] Gardiner, C. & Zoller, P. *Quantum noise: a handbook of Markovian and non-Markovian quantum stochastic methods with applications to quantum optics* (Springer, 2004).

